# Downregulated luteolytic pathways in the transcriptome of early pregnancy bovine corpus luteum are mimicked by interferon-tau in vitro

**DOI:** 10.1186/s12864-021-07747-3

**Published:** 2021-06-16

**Authors:** Raghavendra Basavaraja, Jessica N. Drum, Jackson Sapuleni, Lonice Bibi, Gilgi Friedlander, Sai Kumar, Roberto Sartori, Rina Meidan

**Affiliations:** 1 Department of Animal Sciences, The Robert H. Smith Faculty of Agriculture, Food and Environment, The Hebrew University of Jerusalem, 7610001 Rehovot, Israel; 2 Department of Animal Science, University of São Paulo, Piracicaba, Brazil; 3 The Mantoux Bioinformatics institute of the Nancy and Stephen Grand Israel National Center for Personalized Medicine, Weizmann Institute of Science, Weizmann Institute of Science, 7610001 Rehovot, Israel

**Keywords:** RNA seq, Maternal pregnancy recognition, Prostaglandin F2 alpha, Luteinized granulosa cell

## Abstract

**Background:**

Maintenance of the corpus luteum (CL) beyond the time of luteolysis is essential for establishing pregnancy. Identifying the distinct features of early pregnancy CL remains unresolved, hence we analyzed here the transcriptome of CL on day 18 pregnant (P) and non-pregnant (NP) cows using RNA-Seq. CL of P cows expressed ISGs, verifying exposure to the pregnancy recognition signal, interferon-tau (IFNT), whereas the CL of NP cows had elevated luteal progesterone levels, implying that luteolysis had not yet commenced.

**Results:**

The DEGs, IPA, and metascape canonical pathways, along with GSEA analysis, differed markedly in the CL of P cows from those of NP cows, at the same day of the cycle. Both metascape and IPA identified similar significantly enriched pathways such as interferon alpha/beta, sonic hedgehog pathway, TNFA, EDN1, TGFB1, and PDGF. However, type-1 interferon and sonic hedgehog pathways were positively enriched whereas most of the enriched pathways were downregulated in the P compared to NP samples. Thirty-four % of these pathways are known to be elevated by PGF2A during luteolysis. Notably, selective DEGs in luteinized granulosa cells were modulated by IFNT in vitro in a similar manner to their regulation in the CL of P cows.

**Conclusion:**

This study unraveled the unique transcriptomic signature of the IFNT-exposed, early pregnancy CL, highlighting the abundance of downregulated pathways known to be otherwise induced during luteolysis. These and IFNT-regulated in vitro pregnancy-specific DEGs suggest that IFNT contributes to the characteristics and maintenance of early pregnancy CL.

**Supplementary Information:**

The online version contains supplementary material available at 10.1186/s12864-021-07747-3.

## Background

In mammalian species including cattle, a corpus luteum (CL) that produces and secretes adequate amounts of progesterone is critically important for reproductive success [1–3]. Indeed, there is a direct relationship between the concentrations of progesterone and embryonic survival during early pregnancy [4, 5]. In the absence of an embryonic signal, uterine prostaglandin F2A (PGF2A) luteolytic pulses will cause CL regression [6, 7]. However, if fertilization occurs, the viable embryo will signal its presence so that the CL is maintained [1, 2]. Interferon tau (IFNT) has been shown to be the definitive pregnancy recognition signal in ruminants [8, 9]. It is structurally homologous to the other type-1 interferons such as interferon alpha (IFNA) and interferon beta (IFNB). All of them act through the classical type-1 interferon pathway and stimulate the Interferon-stimulated genes (ISGs) in the endometrium [10–13]. These ISGs include the interferon regulatory factors (IRFs) family, ISG15, MX1 (MX Dynamin Like GTPase 1), MX2, 2′-5′-Oligoadenylate Synthetase 1 (OAS1Y), and signal transducer and activator of transcription (STATs) [14–18]. IFNT in cows is produced by the trophoblastic cells throughout days 7–28 after insemination [8, 19, 20], with peak production on days 18–20 [19]. There is ample evidence that IFNT acts in the endometrium to inhibit uterine pulses of PGF2A [8, 9, 21, 22] during maternal recognition of pregnancy. Nevertheless, in addition to its uterine actions, an endocrine role for IFNT has been suggested [9, 23]. Antiviral activity was detected in uterine vein blood [14, 24] and ISGs were expressed in CL from pregnant ruminants as well as during uterine vein infusion of recombinant ovine IFNT (roIFNT) [14, 17, 18, 25]. Notably, endocrine delivery of roIFNT, via the uterine or jugular vein, protected the ovine CL from the luteolytic actions of PGF2A [26]. Furthermore, intraluteal and circulating progesterone levels as well as CL volume were maintained, and these CL had a greater expression of genes for cell survival [26]. Our in vitro data, which show the effects of IFNT on luteal cells [16, 17, 27], further support a direct role of IFNT on luteal function.

How CL in distinct physiological states differ (i.e., cyclic, regressed, and pregnancy CL), remains an intriguing question. Specifically, it is unclear whether the distinct features of early pregnancy CL are due to reduced PGF2A, the endocrine action of IFNT, or other yet unknown factors.

Several transcriptomic studies of early pregnant ruminant CL have been conducted in recent years [15, 28–30]. These studies reported genes and pathways that are specific for pregnancy. However, despite the wealth of information provided, there was little overlap of the differentially expressed genes (DEGs) and the regulated pathways in these studies. Most probably due to the different reference group, which were cyclic, pregnant at different stages, or regressing CL [15, 28–30]. Here we analyzed the transcriptome of day matched (day 18) CL of pregnant (P) and non-pregnant (NP) cows. Our data show that the CL of P cows expressed ISGs, suggesting an exposure to IFNT, whereas the CL of NP cows had elevated luteal progesterone levels, implying that they had not yet begun luteolysis. Therefore, a comparison of these CL provides unique physiological model to uncover luteal effects conferred by IFNT. To gain further understanding of IFNT actions, selective DEGs identified in the transcriptomic analysis were determined in luteinized granulosa cells (LGCs) incubated in vitro with roIFNT.

## Results

### Transcriptomic comparison of day 18 CL pregnant (P) and non-pregnant (NP) cows

**Fig. 1:**
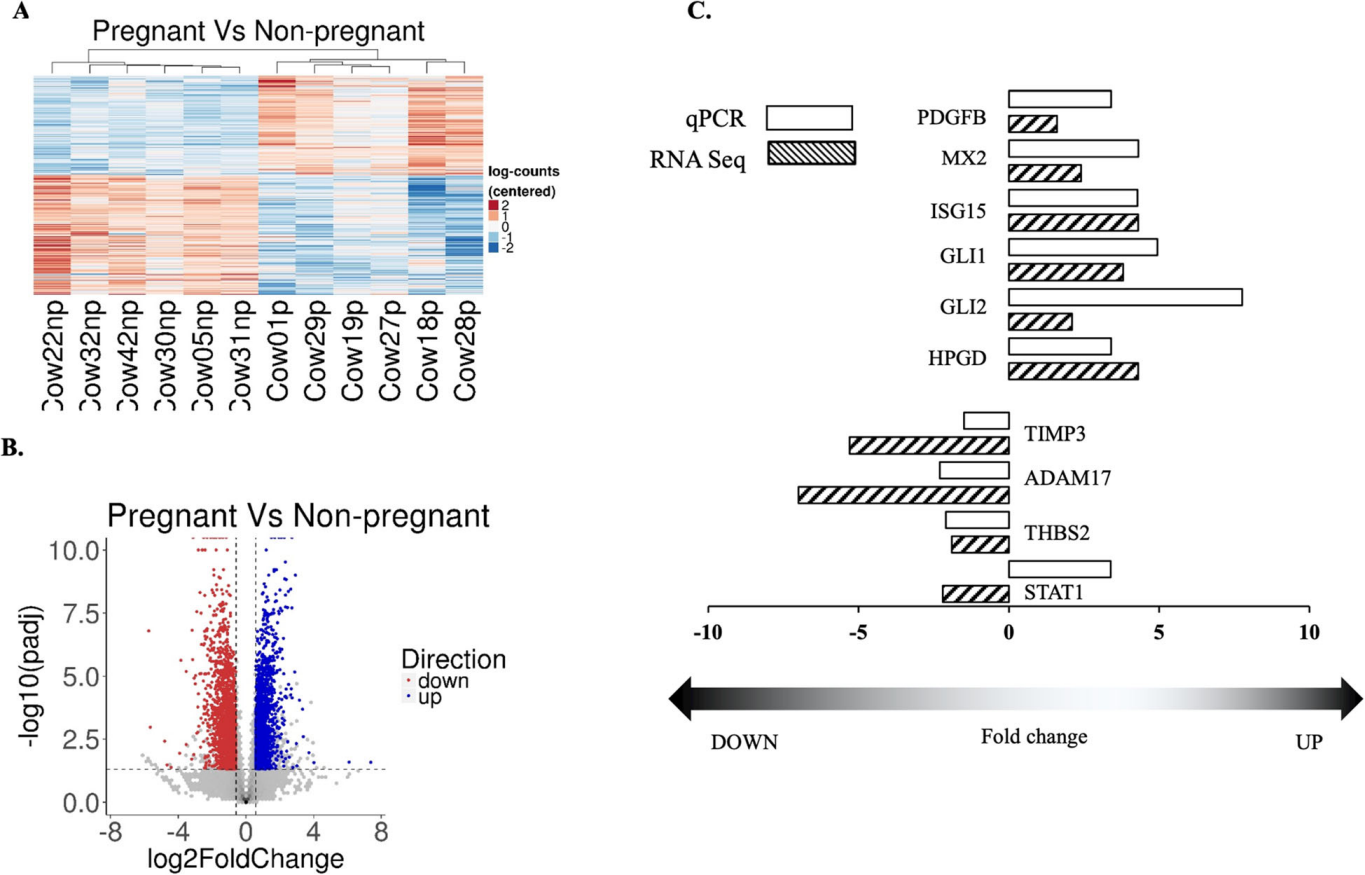
Global changes of DEGs in day 18 P compared to NP bovine CL. (A) A total of 3437 genes were significantly differentially expressed between the CL of P compared to NP samples. The log 2 normalized counts were centered to have for each gene a mean of zero. Hierarchical clustering of the centered values was performed with the Pearson dissimilarity measure. The expression profile is accompanied by a colored bar indicating the centered log 2 normalized counts. Hierarchical clustering of the centered values was performed with the Pearson dissimilarity measure. Each column represents an individual cow and each row represents a gene. (B) A volcano plot was constructed, satisfying the criteria of -log2 fold-change value > 0.58 or < − 0.58 and *padj* < 0.05. The x-axis and y-axis in the volcano plot represent the log 2 ratio and –log 10 (*padj*), respectively. Points are colored according to their average log ratio in the data set. Red and blue indicate that the expression level was decreased or increased, respectively. (C) Validation of selective DEGs with qPCR analysis where the fold change (P compared to NP) of RNA seq analyses (dark patterned bars) was compared with the qPCR results (open bars). Positive and negative values represent up- and down-regulated DEGs, respectively

Prior to tissue collection, pregnancy was confirmed by the presence of an embryo in uterine flushes on the day of slaughter. Luteal progesterone concentrations did not differ between the P and NP cows (*P* = 746 ± 6.7 pg/ug protein; (*n* = 6); NP = 635 ± 190 pg/ug of protein (n = 6), confirming that CL of NP group were not undergoing regression at the time of collection. RNA seq analysis using the NGS platform was carried out in order to identify the transcriptomic changes between day 18 CL of the P and NP group. Initially principal component analysis (PCA) was used to assess the overall mRNA profile differences among the samples; it showed that the P and NP samples were clustered separately (Supplementary Figure 1). The hierarchical clustering was performed along with a dendrogram on top, which is drawn as shown in Fig. 1A. There was a similar expression pattern among the samples and a clear separation of NP from P samples was observed. As shown in Fig. 1B, a volcano plot of differential abundance revealed that there were 3437 DEGs between P compared to NP. Among the 3437 DEGs, 1872 (54%) genes were downregulated, whereas 1565 (46%) genes were upregulated when comparing P and NP (Supplementary Figure 1). For validation of DEGs in RNA seq by qPCR, ten DEGs (*ISG15, MX2, PDGFB,* GLI family zinc finger 1 (*GLI1*), *GLI2*, *HPGD*, *TIMP3*, *ADAM17*, *THBS2*, and *STAT1*) were selected. RNA samples from the same samples assigned to the RNA-Seq analysis (*n* = 6 for either P or NP) were used for the qPCR validation. The pattern of expression observed by qPCR was similar to the RNA seq analysis except for *STAT1*, as depicted in Fig. 1C.

### Functional enrichment and pathway analysis

**Fig. 2:**
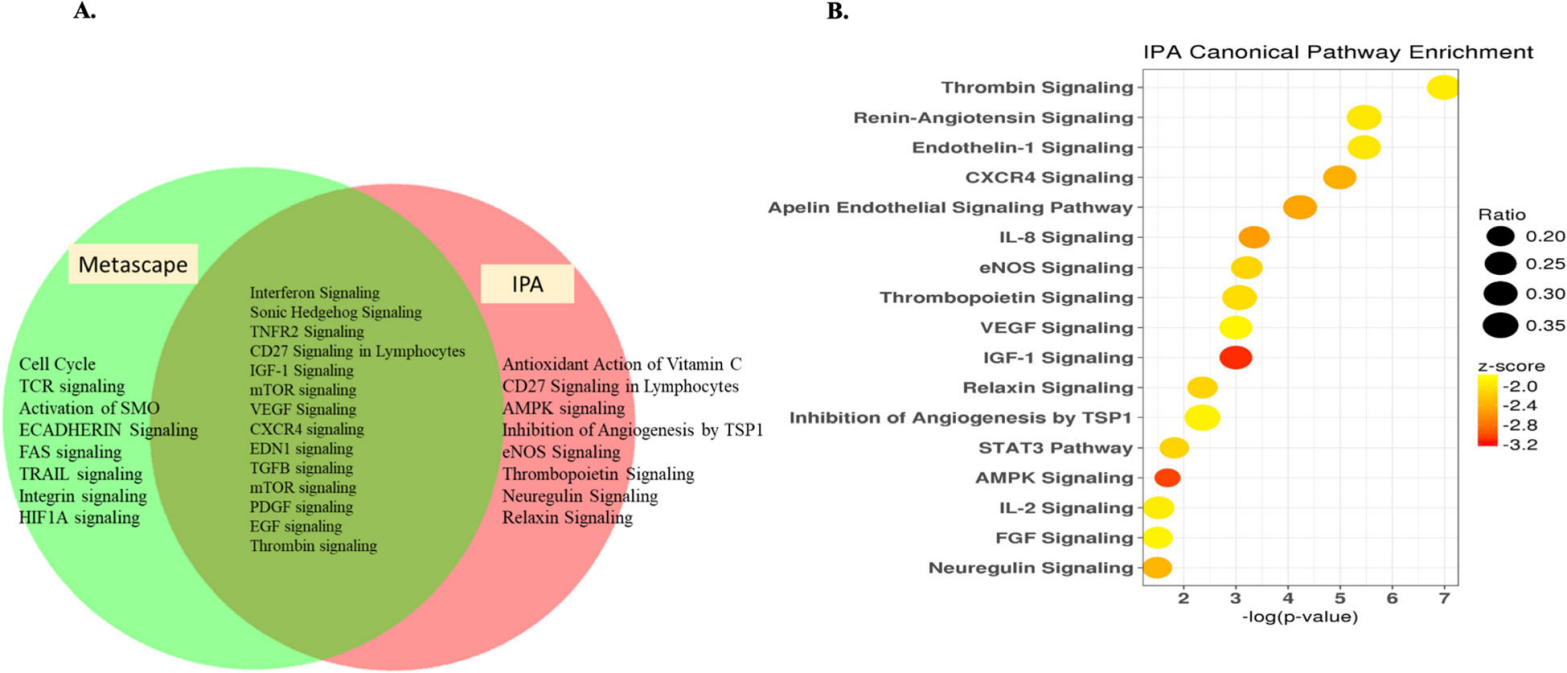
Functional pathway enrichment of DEGs in day 18 P compared to NP bovine CL. (A) Pathway enrichment analysis was performed using Metascape and IPA on both up- and downregulated DEGs. The significantly (*padj* < 0.05) enriched canonical pathways in both tools are represented in a Venn diagram. (B) A dot plot of selected significantly enriched pathways from IPA analysis that were down-regulated in P compared to NP (*padj* < 0.05; Z-score < − 1.9). The x-axis represents the –log *padj*. The color indicates the Z-score (a Z-score below − 2 indicates that the pathway is significantly downregulated). The size of the dots represents the gene ratio (the number of genes in the pathway that were significantly differentially expressed relative to the number of genes in the pathway)

**Fig. 3:**
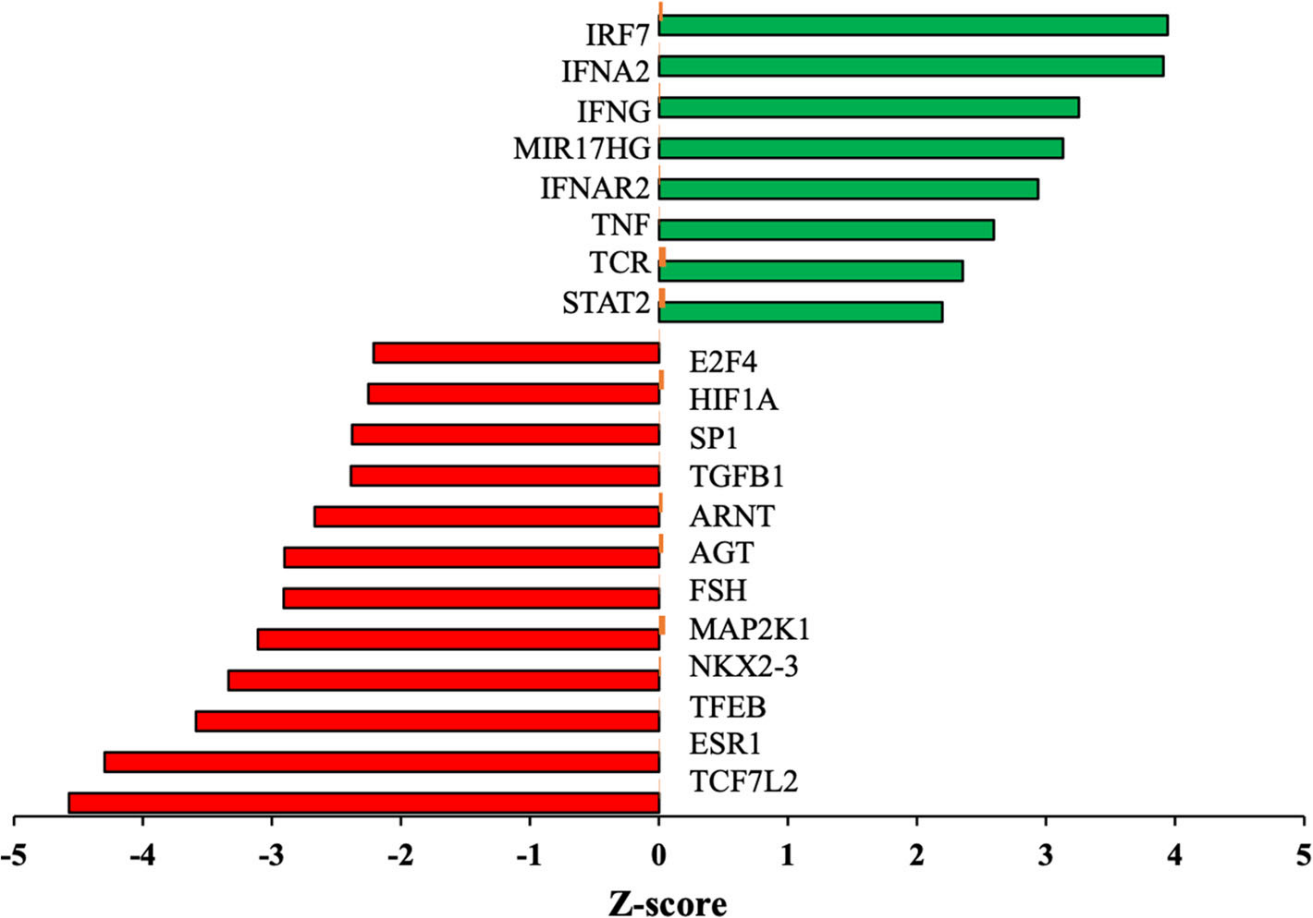
Upstream regulators for DEGs in bovine CL from day 18 P and NP cows. Bar graphs show the top activated and inhibited upstream regulators in day 18 P cows compared to NP cows predicted by IPA analysis (default cut off Z-score > 2 or < − 2 and *padj* < 0.05). Green bars indicate the activated upstream regulators and the red bars indicate the upstream-inhibited regulators. The x-axis represents the Z-score

To gain more insight into key processes that may explain the functional differences between the P and NP group, functional annotation analyses were carried out using IPA and metascape. Both metascape and IPA identified 14 common, significantly enriched pathways such as interferon alpha/beta, sonic hedgehog (SHH), tumor necrosis factor receptor 2 (TNFR2), EDN1, TGFB1, and thrombin (Fig. 2A). Figure 2B presents selective downregulated pathways with a significant inactivation score (Z-score) and *padj* ≤ 0.05 in IPA analysis. Most of the enriched pathways were downregulated in the P compared to NP samples. Then by analyzing the linkage to DEGs through coordinated expression, we identified, using IPA tool, potential upstream regulators [31]. These predictions are based on the literature compiled in the Ingenuity pathway knowledge base. The top upstream regulators (both activated and inactivated) in the P compared to the NP samples data set are listed in Fig. 3 (Z-score ≥ 2 and *pad* ≤ 0.05). There is an apparent overlap in the regulatory actions of these regulators with the affected pathways, illustrated in Fig. 2A and B. Most of the activated upstream regulators include receptors and transcription factors associated with the interferon pathway (IFNAR1, IFNAR2, STAT2, IFNG, and IRF7; Fig. 3). Other activated upstream regulators were TNF, a pleiotropic cytokine and STAT2 that mediates signaling by type I IFNs. The inhibited set of upstream regulators list included ESR1, TGFB1, FSH, ARNT, HIF1A, and others, depicted in Fig. 3.

### Type-1 interferon and sonic hedgehog pathways are positively enriched in the day 18 CL of P compared to NP cows

**Fig. 4:**
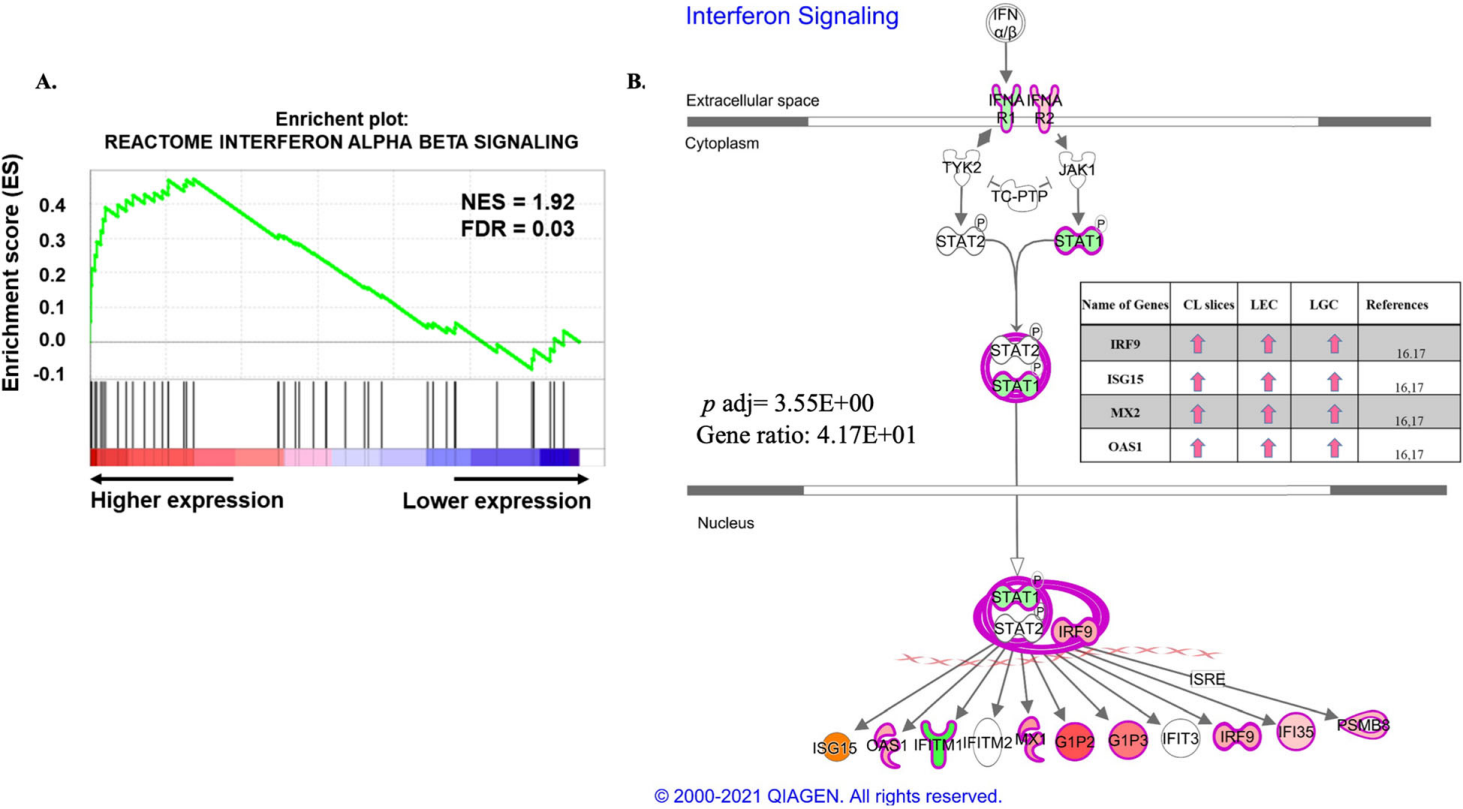
Type-1 interferon pathway of DEGs in P compared to NP samples from day 18 bovine CL clustered by GSEA and IPA. (A) GSEA enrichment plot showing type-1 interferon pathway candidate genes enriched in P compared to NP bovine CL. Normalized enrichment signal (NES); Normalized *p*-value (NOM), False Discovery Rate (FDR). (B) Type-1 interferon pathway generated with IPA. Genes that are significantly up- and downregulated are shown in red and green, respectively. The intensity of the color corresponds to an increase or decrease in fold change between the P and NP bovine CL. The inset table indicates the expression of select ISGs in CL slices and luteal cells treated with roIFNT (1 ng/mL) in vitro, previously published

We also used Gene Set Enrichment Analysis (GSEA) to identify potential pathways that are modified in the early pregnancy CL. GSEA within MSigDB using a stringent false discovery rate (FDR) cutoff (FDR < 0.05) revealed the enrichment of several pathways in the P compared to NP samples. Among them, there was an enrichment of type-1 interferon pathway genes in the P group compared to the NP group (normalized enrichment signal (NES) = 1.92 and false discovery rate (FDR) = 0.034, Fig. 4A). The key genes, included in the list are *MX1*, *ISG15*, *OAS1*, *MX2*, *IRF9*, *IRF3,* and other type-1 interferon-related genes. This analysis is in agreement with data shown in Fig. 2A and 3. Furthermore, IPA network analysis also showed that type-1 interferon pathways were significantly enriched (*padj* = 0.003) with genes including *ISG15*, *OAS1*,*MX1*, *GIP2*, *GIP3,* and *IRF9* (Fig. 4B). Some of these ISGs (*IRF9*, *ISG15*, *MX2,* and *OAS1*) were previously shown to be significantly elevated by in vitro treatment of roIFNT in bovine CL tissue, luteal endothelial cells, and LGCs [16, 17] (Insert table Fig. 4B).

**Fig. 5:**
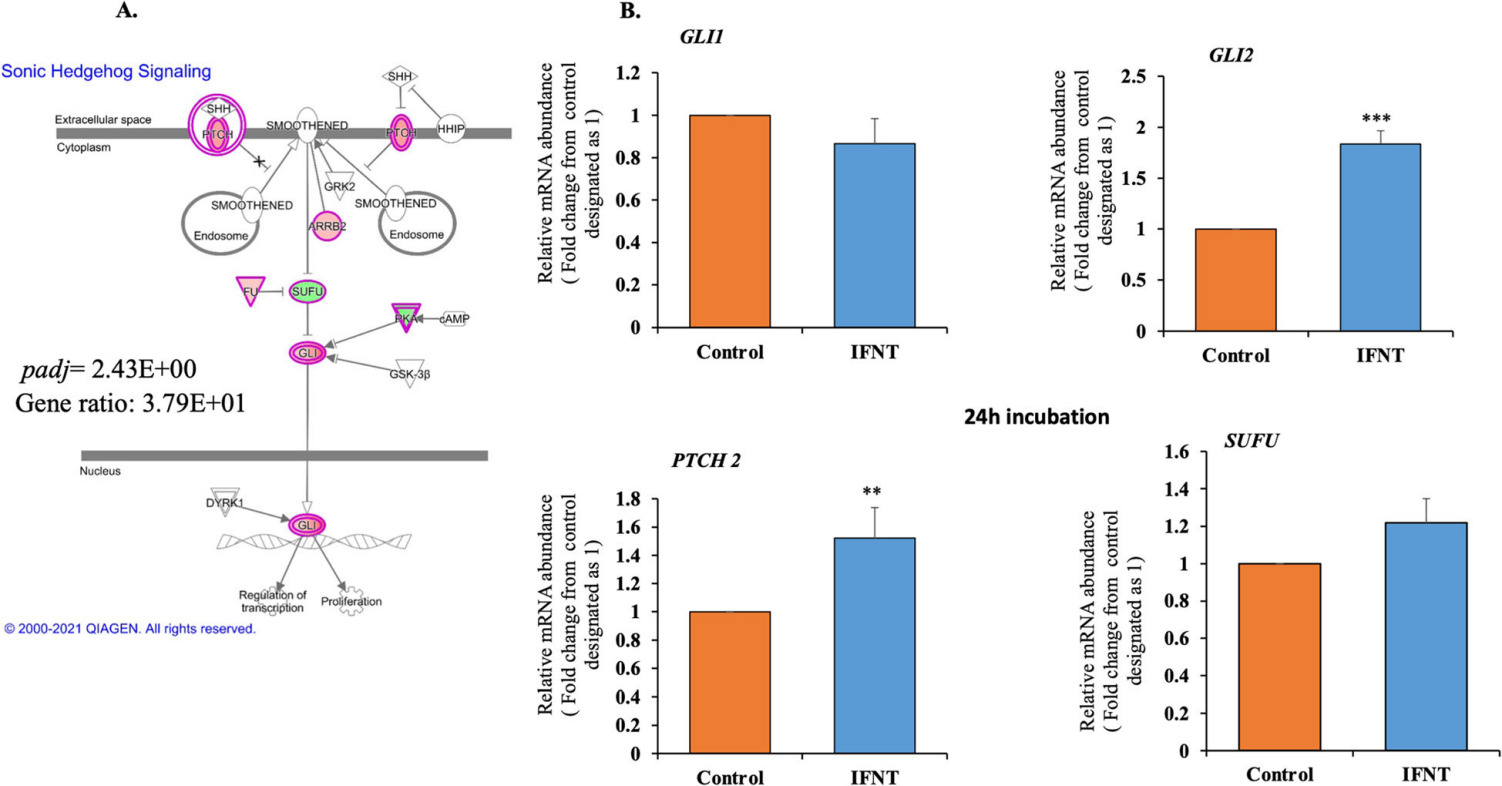
Sonic hedgehog pathway in bovine CL and LGCs. (A) Sonic hedgehog pathway from IPA, genes that are significantly up- and downregulated are shown in red and green, respectively. The intensity of red and green colors corresponds to an increase or decrease in fold change levels of P compared to NP bovine CL, respectively (B) LGCs (*n* = 5) were treated with either basal media (control) or roIFNT (1 ng/mL) for 24 h. *GLI1*, *GLI2*, *PTCH2*, and *SUFU* mRNA expression were measured by qPCR. Asterisks denote significant differences from their controls (**p < 0.01 and***p < 0.001)

Sonic hedgehog is one of the few prominent pathways that were significantly activated in P compared to NP samples in both the metascape and IPA analysis. Differentially regulated candidate genes, along with their pathways exported from IPA, are shown in Fig. 5A. Among the candidate genes, GLI1, GLI2, and PTCH2 were significantly upregulated in the P compared to NP samples. Inhibitors of the SHH pathway such as the SUFU and PKA family were negatively regulated in the P compared to NP samples (Fig. 5A). To assess the effect of in vitro roIFNT treatment on this pathway, LGCs were cultured for 24 h with 1 ng/mL of roIFNT. As shown in Fig. 5B, roIFNT significantly upregulated *GLI2* and *PTCH2* without affecting *GLI1* and *SUFU* expression.

### Pregnancy negatively enriched TGFB and matrix metalloproteinase pathways in day 18 CL

**Fig. 6:**
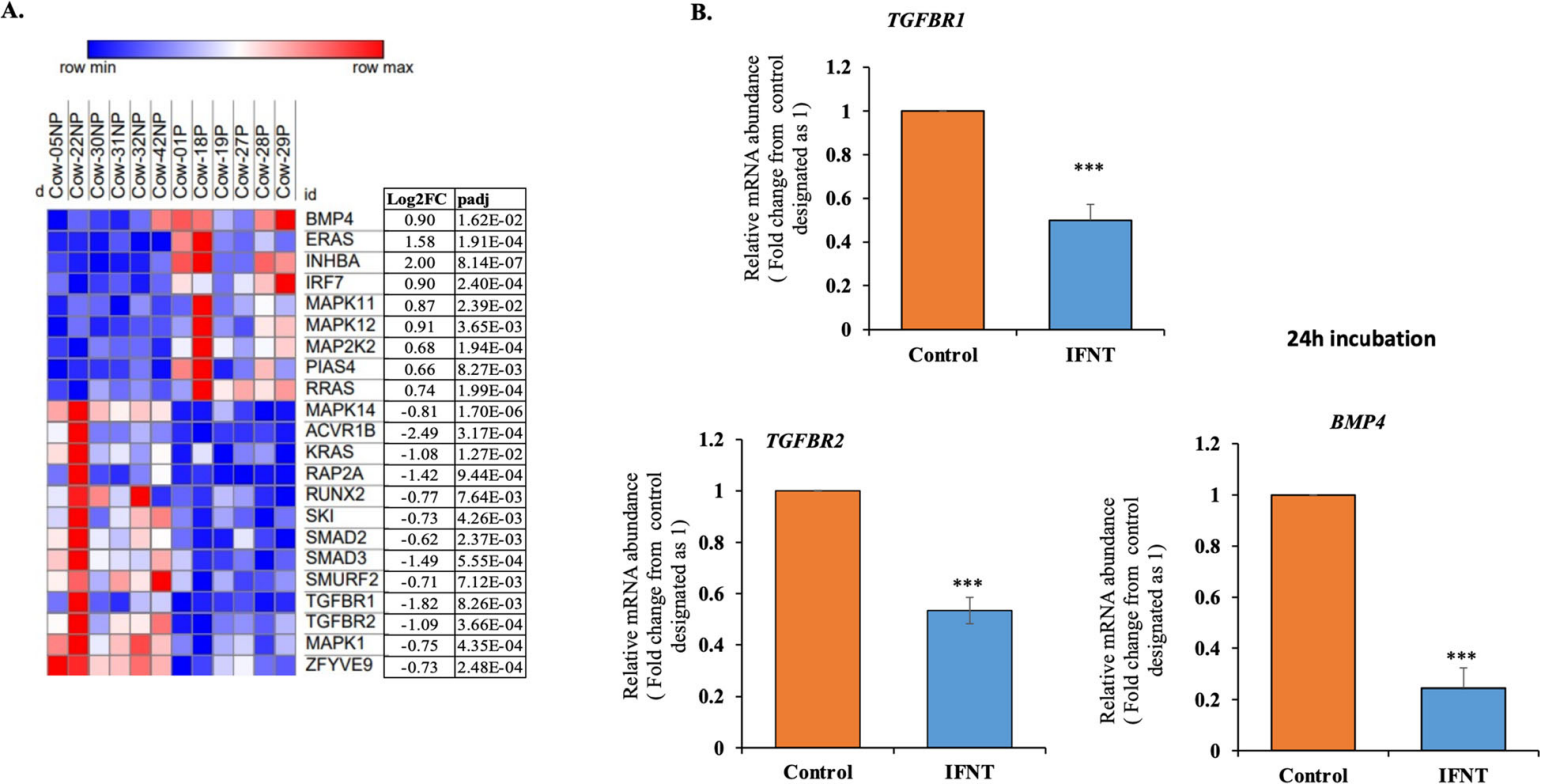
Analysis of the TGFB pathway in the P compared to NP bovine CL and the in vitro effect of IFNT in LGCs. (A) Heatmap showing differentially abundant TGFB pathway candidate genes. The heatmap was generated by Morpheus - Broad Institute (https://software.broadinstitute.org/morpheus/). Higher and lower levels of DEGs are denoted in red and blue, respectively, and the median level is denoted in white. The intensity of color corresponds to an increase or decrease in the fold change of the particular DEG. (B) LGCs (*n* = 4) were treated with either basal media (control) or roIFNT (1 ng/mL) for 24 h. *TGFBR1*, *TGFBR2* and *BMP4* expression were measured by qPCR. Asterisks denote significant differences from their controls (***p < 0.001)

The IPA analysis revealed that candidate genes related to the TGFB pathway were significantly regulated, as illustrated by a heat map in Fig. 6A. Within this group of DEGs, a subset of 13 genes was significantly downregulated including *TGFBR1*, *TGFBR2* and their signaling molecules, *SMAD2* and *SMAD3* (Fig. 6A), whereas 9 genes were upregulated in the P compared to NP samples. This list included *BMP4*, *ERAS, INHBA,* and others. Similarly, to P cows, the in vitro results show that roIFNT inhibited markedly, by almost half, the expression of the two TGFB1 receptors, *TGFBR1* and *TGFBR2*, compared with the control (Fig. 6B). However, contrary to P cows, roIFNT significantly downregulated the expression of *BMP4* in LGCs (Fig. 6B).

**Fig. 7:**
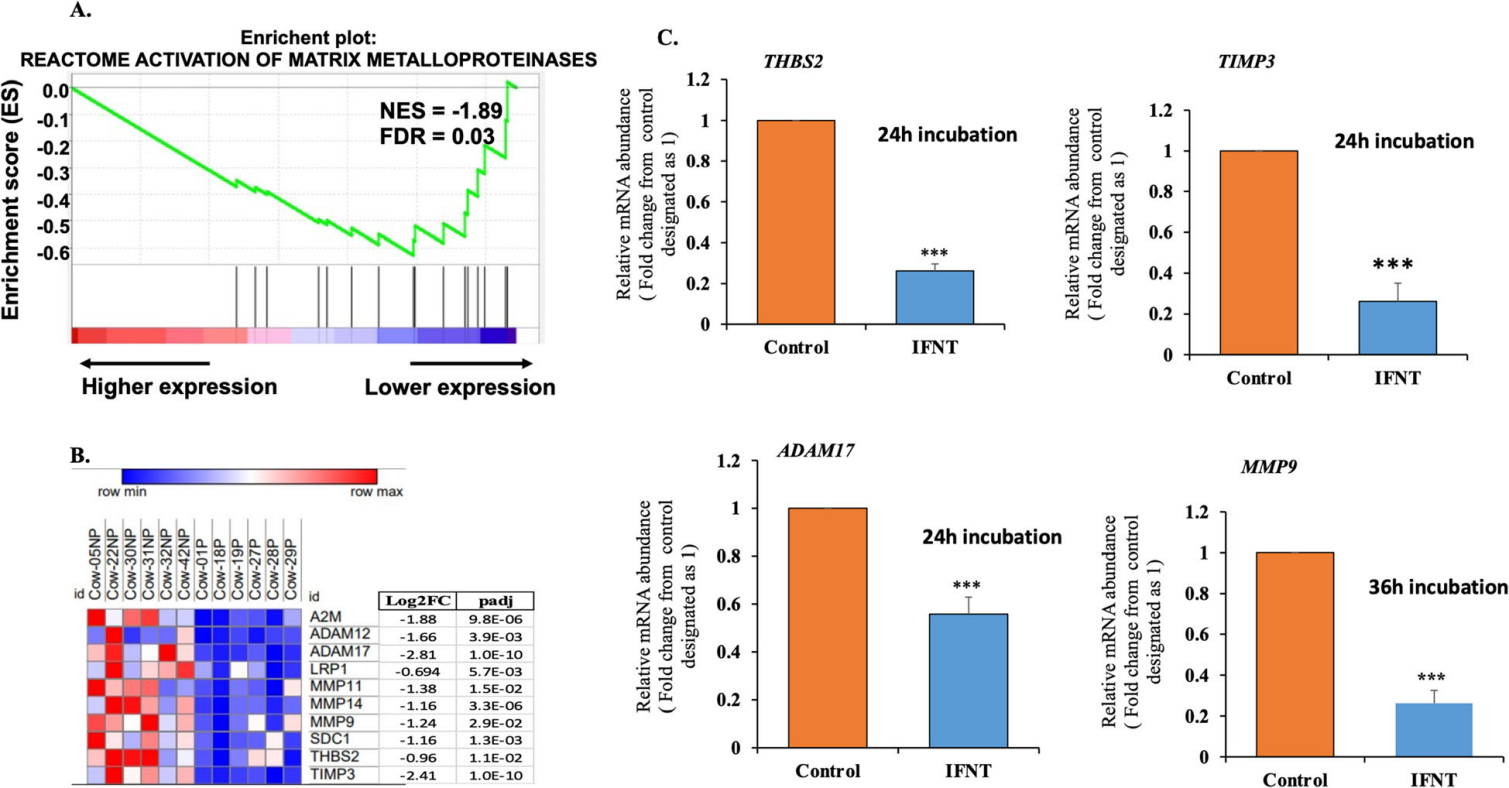
Matrix metalloproteinases pathway in P compared to NP of day 18 bovine CL and the in vitro effect of IFNT in LGCs. (A) GSEA results showing the activation of matrix metalloproteinase candidate genes enriched in day 18 P compared to NP bovine CL. (B) Heatmap showing the differentially abundant activation of matrix metalloproteinase candidate genes. The heat map was generated by Morpheus - Broad Institute (https://software.broadinstitute.org/morpheus/). Higher and lower levels of transcript accumulation are denoted in red and blue, respectively, and the median level is denoted in white. (C) LGCs (n = 5) were treated with either basal media (control) or roIFNT (1 ng/mL) for 24 h for all except *MMP9*, which was for 36 h. *THBS2*, *TIMP3*, *ADAM17*, and *MMP9* expression was measured by qPCR. Asterisks denote significant differences between roIFNT treatment and controls (***p < 0.001)

As shown in Fig. 7A, GSEA analysis revealed that the activation of the matrix metalloproteinase (MMP) pathway is negatively enriched in P compared to NP cows with NES = − 1.9 and FDR < 0.033. The genes related to this pathway are also illustrated by a heat map, as depicted in Fig. 7B. To study whether some of the genes related to this pathway are affected by IFNT, we determined their expression in LGCs that were treated with roIFNT. The results indicated that *THBS2*, *TIMP3*, *ADAM17*, and *MMP9* were all significantly downregulated at 24 h incubation expect *MMP9* which required a longer incubation period (36 h; Fig. 7C).

**Fig. 8:**
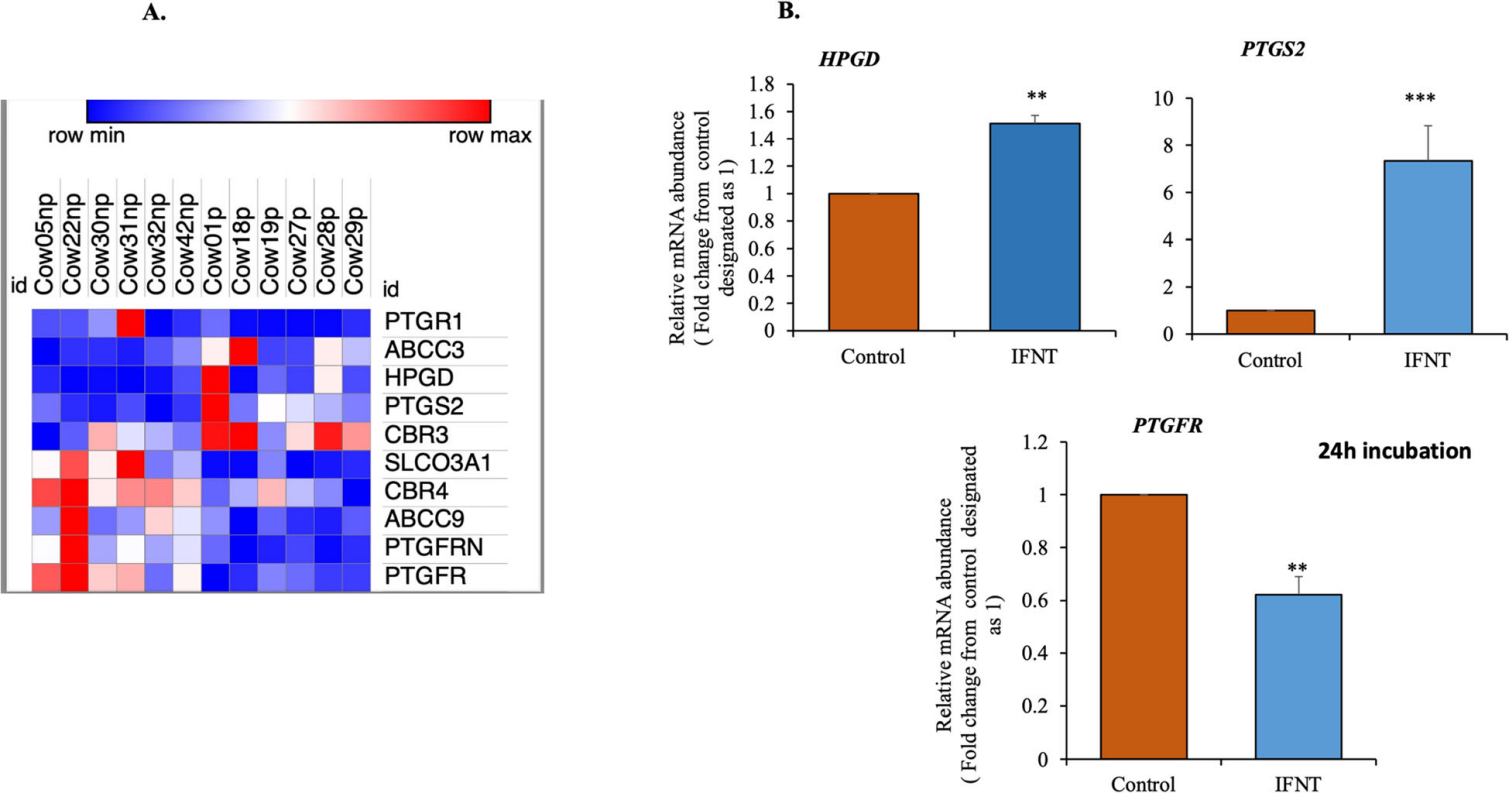
Selective DEGs related to prostaglandin response and metabolism in day 18 P compared to NP bovine CL and the in vitro effect of IFNT in LGCs. (A) Heatmap showing the differentially abundant genes that are related to prostaglandin response and metabolism and that were significantly differentially expressed. The heat map was generated by Morpheus - Broad Institute (https://software.broadinstitute.org/morpheus/). Higher and lower levels of transcript accumulation are denoted in red and blue, respectively, and the median level is denoted in white. The log 2-fold change and the *padj* for a comparison of pregnant versus non-pregnant cows in the RNA Seq is shown. (B) LGCs (n = 5) were treated with either basal media (control) or roIFNT (1 ng/mL) for 24 h. *HPGD*, *PTGS2* and *PTGFR* expression were measured by qPCR. Asterisks denote significant differences between roIFNT treatment and controls (**p < 0.01 and ***p < 0.001)

Among the DEGs, we noted that genes associated with prostaglandin response and metabolism, e.g., 5-Hydroxyprostaglandin Dehydrogenase (HPGD), Prostaglandin-endoperoxide synthase (PTGS2), and the oxidoreductase family gene, CBR3, were positively regulated in the P compared to NP samples as depicted in Fig. 8A heatmap. Whereas the PGF2A receptor, PTGFR, the receptor inhibitor, *PTGFRN*, its transporter*, ABCC9*, and others were negatively regulated in P compared to NP samples (Fig. 8A heatmap). In LGCs, roIFNT mimicked some of these effects, for instance, the expression of *HPGD* (~ 1.3-fold), and *PTGS2* (~ 8-fold) were elevated, whereas PTGFR was downregulated, as shown in Fig. 8B.

**Fig. 9:**
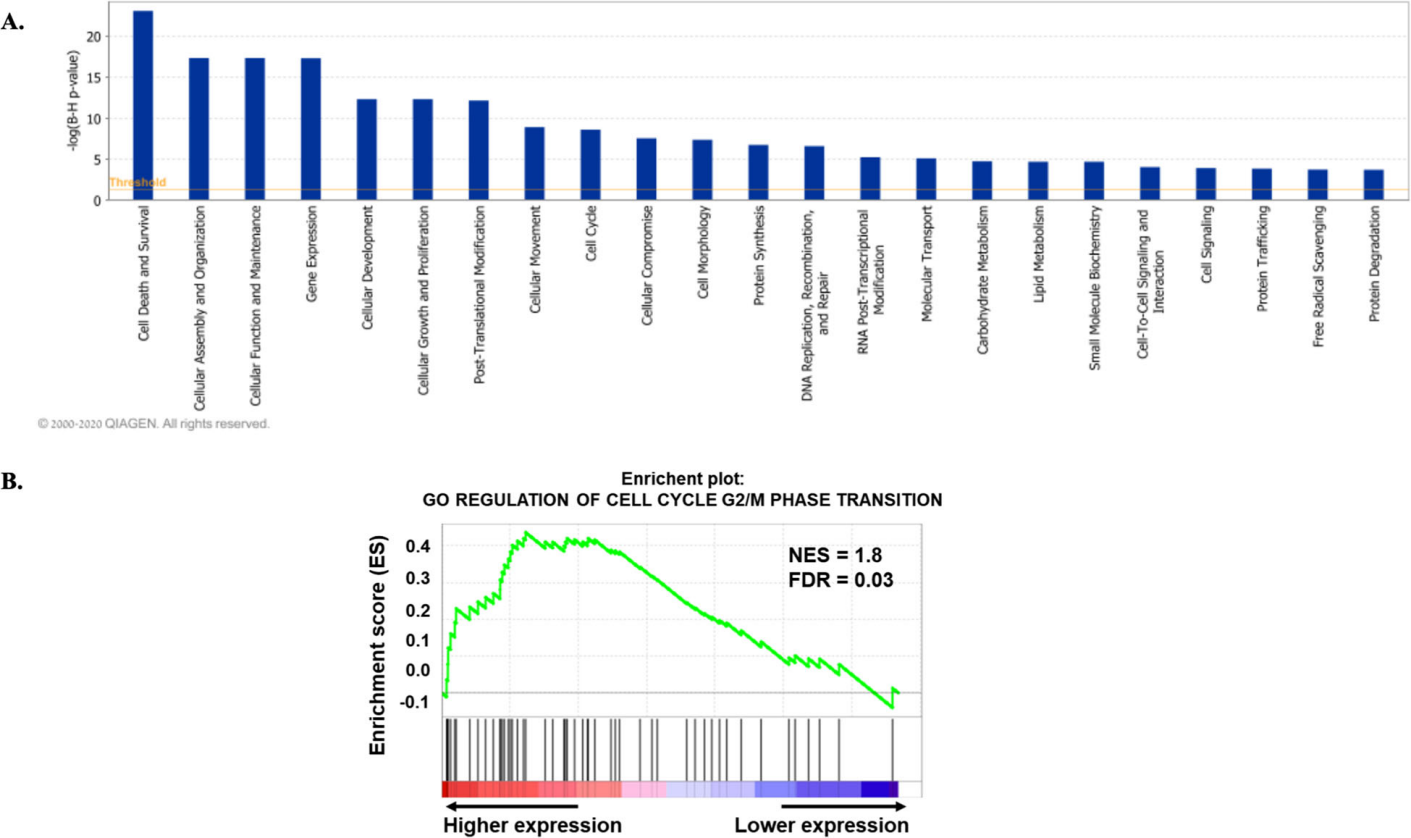
Enrichment of biological functions related to cell cycle in P compared to NP day 18 bovine CL. **A** Bars indicate the likelihood [−log B-H (p-value)] that the specific molecular and cellular functions obtained from IPA disease and biological functional categories analysis was affected by P cows compared with NP cows. **B** GSEA enrichment plot of the Gene Ontology (GO) term regulation of the cell cycle G2/M transition

The IPA diseases and biological functions tool identified several networks associated with cellular and molecular functions, which include cell death and survival, cellular function and maintenance, cellular proliferation and survival, cellular movement as well as the cell cycle (Fig. 9A). Further analyses utilizing GSEA GO terms, which incorporate the directionality of gene changes between groups, revealed positive enrichment of cell cycle of G2-M transition in P compared to NP groups (Fig. 9B).

## Discussion

This study utilized various functional annotation tools to analyze the transcriptome of bovine CL, dissecting the molecular profile of d18 P cows for the first time and demonstrating that it is strikingly different from that of non-luteolytic, NP CL at the same day of the cycle. This conclusion is based on DEGs, IPA, and metascape canonical pathways, GSEA enrichment plots, along with molecular and cellular functions. Unlike previous publications, this study captured the transcriptome of early pregnancy CL exposed to IFNT and compared it to non-luteolytic cyclic CL, thus singling out the effects of IFNT [15, 28–30].

Several findings here indicate that the CL of P cows were exposed to IFNT, including the enrichment of the IFNA/B signaling pathway, IFN and its receptors were identified as an upstream regulators along with various ISGs differentially expressed in the P compared to NP analysis. An outstanding finding of this study was that most IPA canonical pathways were downregulated in P cows, whereas very few pathways were upregulated. Importantly, the selective DEGs studied here in LGCs, were regulated by roIFNT in vitro similarly to their regulation in the CL of P cows, supporting the notion that IFNT affects luteal gene expression in vivo*.* This is further corroborated by previous findings demonstrating that various ISGs (*IRF9*, *ISG15*, *MX2,* and *OAS1*) were elevated by roIFNT treatment in vitro of in various luteal cell models [16–18, 32, 33].

Many of the downregulated pathways in the pregnancy CL were pathways whose involvement in luteal regression was established before. Among them EDN1, eNOS, CXCR4, VEGF, IL8, TGFB1, THBS1, IGF1, STAT3, and FGF2 signaling [15, 16, 34–38]. Moreover, most of these pathways were previously shown to be induced by PGF2A specifically in the responsive gland [7, 35, 38, 39]. The suppression of the luteolytic pathway in the pregnancy CL suggested by our findings imply that at this initial phase of pregnancy the gland can resist PGF2A luteolytic actions. Our results provide an explanation why in ruminants ample evidence showed that the CL of early pregnancy is more resistant to the luteolytic action of PGF2A [40–46].

The issue of whether and how PGF2A secretion differs in the pregnant compared the cyclic cow or ewe is controversial. On the one hand, pregnant ruminants were reported to have higher concentrations of basal PGF2A than cyclic animals have [47, 48]. However, recent publications have suggested that although the numbers of PGF2A pulses per day were significantly higher during luteolysis, compared with pregnant animals, they were similar in the pregnant animals and in those at the late luteal phase [40, 49]. Additionally, PGF2A can reach the CL during early pregnancy as shown by Banu and coworkers [50].

TGFB1 signaling is a pathway that was shown to be significantly enriched and repressed in the current study in P compared to NP cows, as well as in previous reports [15, 28] (see Figs. 2, 3, and 6). TGFB1 is extensively studied in relation to luteolysis and was shown to be specifically upregulated by PGF2A in the mature CL [34, 51]. Functionally, TGFB1 promotes apoptosis of bovine luteal endothelial cells [51–53], limits their cell growth, and disrupts capillary morphogenesis and endothelial barrier function [53]. Previously we reported that IFNT inhibits *TGFB1* expression in luteal endothelial cells and CL slices [16], here we observed that *TGFBR1* and *TGFBR2* were inhibited in vitro by IFNT in LGCs, suggesting a multicellular, effective shutdown of TGFB1 signaling in the pregnancy CL.

Downregulation of TGFB1 signaling in the CL of P cows would preserve the structure and function of luteal vasculature. Similarly, inhibition of THBS1 proapoptotic and antiangiogenic activities [51, 54] during pregnancy is expected to support luteal blood vessel integrity. Another factor that may take part in vascular maturation is PDGFB, which attracts pericytes, thus stabilizing blood vessels [55]. This study demonstrated that *PDGFB* is upregulated in the CL of P cows and is stimulated by roIFNT in LGCs in vitro [17]. Corroborating this notion, in bovine luteal endothelial cell network established in vitro, the inhibition of PDGF signaling markedly decreased its formation and sprouting [56]. Notably, FGF2 and VEGFA signaling, the principal mitogens of luteal angiogenesis, were found here to be reduced in early pregnancy, suggesting that there is no new wave of angiogenesis during CL maintenance. Indeed, staining of the endothelial cell marker von Willebrand factor did not change in corpora lutea from day 16 of the estrous cycle to day 40 of pregnancy and remained the same in day 16 P compared to NP cows [55].

EDN1, a vasoactive peptide, was one of first compounds reported to be elevated by PGF2A in a temporal manner during luteolysis [57, 58]. Therefore, inhibition of EDN1 by pregnancy CL (the current study and [59, 60]) or by roIFNT in vitro [16] is expected to maintain dilated vessels that are likely to enable nutrient and hormone exchange. Besides its role as a vasoconstrictor, luteal EDN1 was shown to act as the local mediator of PGF2A in reducing progesterone output [57, 58]. The same applies to renin-angiotensin signaling. Previous studies pointed out that angiotensin II (Ang II) release is stimulated by PGF2A and that Ang II decreases progesterone release [61]. Ang II also acts in synergism with EDN1 during luteolysis [61]. Notably, no major steroidogenic genes were differentially expressed in P cows here and in Romero’s transcriptional analysis of pregnant sheep CL [15]; nevertheless, reduced EDN1 and Ang II can help maintain progesterone output during early pregnancy.

In addition to genes that form luteolytic signature (*TGFB1, THBS1, EDN1*, and *NOS3*), several genes related to prostaglandin metabolism were differentially expressed in pregnancy CL and modulated in vitro by roIFNT. Among those genes related to prostaglandins, *PTGS2* and *HPGD* were upregulated and *PTGFR* was downregulated. Such regulation may imply reduced response to PGF2A and enhanced prostaglandin synthesis during pregnancy most probably PGE2, shown to play luteoprotective role [62, 63].

The current study also unearthed a novel pathway, one of the few upregulated (Z-score = 3.162;*padj* = 0.002) in early pregnancy CL, known as the SHH pathway. It is a highly conserved pathway activated when the SHH ligand binds to its PTCH1 or PTCH2 receptors, relieving the inhibition of smoothened and activated GLI1, GLI2, and GLI3. The transcriptional factors of the GLI family in mammals, including GLI1, GLI2, and GLI3 serve important roles in the SHH signaling pathway. SHH receptor binding also prevents inhibition by the downstream negative regulators SUFU and PKA [64, 65]. Interestingly, the SHH ligand was not differentially expressed in this study. These findings suggest that there is a non-canonical upregulation of the SHH pathway, as reported in previous studies [66, 67]. Future studies should focus on elucidating which compounds can lead to increased expression of the GLI transcription factors in the CL. Antiapoptotic proteins such as BCL-2, MCL-1, and BCL-XL were shown to be GLI target genes [18]. These proteins were increased in CL by IFNT in vivo [15] and in LGCs in vitro [17]. Interestingly, we found here that IFNT elevated *GLI2* and *PTCH2* mRNA expression but not *GLI1* and *SUFU* in LGCs. These findings suggest a possible role for *GLI2* in the survival of luteal cells by the upregulation of anti-apoptotic proteins.

Inactivation of the MMP pathway is enriched in the P cow CL (Fig. 7A) compared with NP CL. MMPs and and their tissue inhibitors (TIMPs) have been implicated as key regulators in the structural involution of the CL during luteal regression [68–70]. PGF2A stimulated MMPs involved in the breakdown of ECM during luteolysis, which accelerates cell detachment from the CL during luteolysis [69, 70]. THBS2, another component of the MMP pathway is upregulated in vivo and in vitro by PGF2A treatment [35, 38] and inhibited during the early period (this study) or for the entire pregnancy [71]. Importantly, the current study shows that IFNT directly inhibited *THBS2* in LGC*,* along with *MMP9*, *ADAM17,* and *TIMP3*, genes constituting the canonical IPA pathway termed “inhibition of MMPs” These results suggest that inhibition of the MMP pathway can serve as a tool to achieve ECM stabilization during pregnancy.

## Conclusions

This study unraveled the distinct molecular profile of early pregnancy CL on day 18, suggesting that it differs substantially from the non-pregnant gland on the same day. Our results suggest that a wide range of functional pathways may be affected: endothelial activation is restrained, blood vessels are stabilized, apoptotic mechanisms are inhibited, steroidogenesis is maintained, and the ECM is preserved. Additionally, the similarity between the regulation of genes by IFNT in vitro and the expression of these genes in the pregnancy CL suggests that the endocrine actions of IFNT may contribute to the characteristics of early pregnancy CL.

## Materials and methods

### Animals and samples collection

The experiment was conducted in accordance with relevant guidelines and regulations at the Experimental Station Hildegard Georgina Von Pritzelwiltz, located in Londrina, PR, Brazil. The Animal Research Ethics Committee of Escola Superior de Agricultura “Luiz de Queiroz” (ESALQ)/University of São Paulo approved all procedures involving cows in this study (Protocol #2018.5.1252.11.5). Non-lactating, *Bos indicus*, multiparous cows (*n* = 35), were submitted to the fixed-time AI (FTAI) protocol as described by Madureira et al. [72]. On the day of insemination (Day 0), cows were assigned into the groups: artificial insemination [AI] group (*n* = 20) or synchronized, non-inseminated group (cyclic; *n* = 10), cows from the AI group were then inseminated using frozen/thawed semen from two high fertility Aberdeen Angus bulls (Alta Genetics, Uberaba, Brazil). On day 17 after AI and 1 day before CL collection, blood samples were collected by puncturing the coccygeal vein into evacuated 10 mL tubes containing sodium heparin (Vacutainer, Dickinson, Franklin Lakes, NJ, USA). Immediately after collection, the tubes were placed on ice and kept refrigerated until processing. Blood samples were centrifuged at 1700×g for 15 min and aliquots of plasma were frozen and stored in duplicate at − 20 °C until they were assayed for progesterone. On Day 18 all cows were slaughtered, and each uterine horn of cows in the AI group was flushed with 10 mL of sterile saline solution. Pregnancy was confirmed by identifying an elongated embryo in uterine flushes, ISGs expression and serum P4 concentration 7 cows were chosen. The presence of a functional CL was confirmed by measuring plasma progesterone using a commercial RIA kit (CT Progesterone, MP Biomedicals LLC, Solon, OH, USA), following the manufacturer’s instructions as previously described and validated for bovine plasma [73]. The intra- and inter-assay CVs were 2.1 and 2.2% respectively, and the sensitivity was 0.05 ng/mL. The CL were collected in cryotubes and immediately frozen in liquid nitrogen and stored at − 80 °C for subsequent RNA extraction and determination of luteal progesterone. Approximately 20 mg of the CL sample, were minced and the homogenates divided into two parts. One part was submitted to a progesterone RIA assay as described above, and the second part of the homogenate was used for protein determination (using Bicinchoninic Acid Kit, Sigma-Aldrich).

### RNA isolation and transcriptomics

For next generation sequencing (NGS), total RNA was isolated from all 14 samples (7 samples for each P and NP groups) using the Tri-Reagent (Molecular Research Center Inc., Cincinnati, Ohio, USA) according to the manufacturer’s instructions. The quantity, quality, and purity of RNA were determined using a 2100 Bioanalyzer instrument (Agilent Technologies, Waldbronn, Germany) and a NanoDrop Spectrophotometer (Thermo Fisher Scientific, Waltham, MA, USA). Only samples with an RNA integrity number (RIN) number greater than 7.5, with all A260/230 ratios > 2.00, and all 260/280 ratios > 1.98 were eligible for this study. Based on the quality and quantity of RNA further analysis were carried out with 6 cows per group. Sequencing and bioinformatics analysis were carried out at The Nancy and Stephen Grand Israel National Center for Personalized Medicine of The Weizmann Institute of Science, Israel.

#### Sequencing

First, 500 ng of total RNA for each sample was processed using an in-house poly A-based RNA seq protocol [74]. Briefly, total RNA was fragmented, followed by reverse transcription and second strand cDNA synthesis. The double strand cDNA was subjected to end repair, A-base addition, adapter ligation, and PCR amplification to create libraries. Libraries were evaluated by Qubit and TapeStation. Sequencing libraries were constructed with barcodes to allow multiplexing. Single end 84 bp reads were sequenced on an Illumina NextSeq 500 machine, yielding a median of 17 M reads per sample. Both before and after the trimming, the median of the first bases was 32 and from base 6 the median of the quality was 36. 92–93% of the reads had a mean quality of at least 30, after the trimming (before the trimming, 90–93% of the reads had a mean quality of at least 30). Most reads had a GC content of 44–45%.

#### Bioinformatics

Poly-A/T stretches and Illumina adapters were trimmed from the reads using cutadapt; resulting reads shorter than 30 bp were discarded. Reads were mapped to the reference genome ARS-UCD1.2 using STAR [75], supplied with gene annotations downloaded from Ensembl (with the End-To-End option). Expression levels for each gene were quantified using htseq-count [76], using the gene transfer format (GTF) file. Differentially expressed genes were identified using DESeq2 [77] with the betaPrior, cooksCutoff, and the independent filtering parameters set to False. Raw *P* values were adjusted for multiple testing using the procedure of Benjamini and Hochberg. The full pipeline was run using snakemake [78]. Genes were considered to be differentially expressed in P compared to NP, with log2 fold change (FC) ≥ 0.58 for the upregulated genes and ≤ − 0.58 for the downregulated genes with the adjusted *p*-value below 0.05 and the gene had a count of at least 30 in one sample. Functional and pathway analysis was performed on transcriptomic data using Ingenuity pathway analysis (IPA), Metascape, and GSEA. Prior to the pathway analysis, DEGs were converted into human orthologues to make use of well-established human pathway analysis databases (KEGG and Reactome).

### Ingenuity pathway analysis (IPA)

IPA (Qiagen, Inc., Valencia, CA, USA) was used to identify the predicted canonical pathways, associated diseases, and functions, key upstream regulators, and the related signaling pathways (https://www.qiagenbioinformatics.com/products/ingenuity-pathway-analysis). A list of DEGs with associated log2FC ≥ 0.58 for the upregulated genes and ≤ − 0.58 for the downregulated genes with *padj* ≤ 0.05 and a count of 60 at least in one of the samples, was uploaded into the IPA server (http://www.ingenuity.com; version 2.3) to identify the enriched canonical pathways. Based on the information stored in the Ingenuity Knowledge Base (IKB), genes were mapped to networks and pathways. Raw *p* values were adjusted for multiple testing using the procedure of Benjamini and Hochberg. Pathways with [−log 10 (B-H (p-value)] > 1.3 (the equivalent of *p* < 0.05) were considered as significantly enriched, along with an activation score (Z-score) above ≥1.90.

### Metascape analysis

Metascape (http://metascape.org/), a gene annotation and analysis resource [79], was used to perform a functional enrichment analysis, which included a canonical, KEGG, and reactome pathway analysis of the overlapping DEGs in all three databases. DEGs that were significantly upregulated (log2FC ≥ 0.58) genes and downregulated (log2FC ≤ − 0.58) genes with padj ≤0.05 were uploaded in a separate list in the Metascape server.

### Gene set enrichment analysis (GSEA)

Whole expression data from the P and NP groups were subjected to gene set enrichment analysis using GSEA [80]. GSEA ranks the genes in expression dataset and then analyzes those pre-ranked genes to determine whether a priori defined sets of genes show statistically significant, concordant differences between two biological states. The differential expression data were tested against the hallmark gene set collection from the molecular signature database, MsigDB v7.2 as well as the KEGG and REACTOME databases. GSEA was applied to the human ortholog genes that were obtained by HomoloGene database and bovine Ensembl. For genes where there was more than one ortholog, one was chosen randomly.

### Isolation and culture of granulosa cells

We collected bovine ovaries bearing large follicles (> 10 mm in diameter) from a local slaughterhouse, as described previously [17, 81]. Only follicles containing at least 4 million granulosa cells were included in these experiments. Granulosa cells were enzymatically dispersed and seeded for overnight incubation in DMEM/F-12 containing 3% fetal calf serum (FCS). The next day, the media was replaced with luteinization media containing FCS (1%), insulin (2 μg/mL), and forskolin (10 μM) [17, 81]. Then, the cells were washed with phosphate buffered saline (PBS), and kept for a 3–5 h adaptation period in DMEM/F-12 media containing 1% FCS. Finally, the cells were incubated 24-36 h as indicated in the results section, with either basal media (containing 1% FCS; control) or roIFNT (1 ng/mL; a generous gift from Prof. Fuller W. Bazer, Texas A&M University and it was functionally validated in numerous studies [9]). At the end of the incubation period, cells were collected for either total RNA extraction, as described below.

### RNA extraction and quantitative real-time PCR

Total RNA was isolated using Tri-Reagent (Molecular Research Center Inc., Cincinnati, Ohio, USA) according to the manufacturer’s instructions. 1 μg of total RNA was reverse transcribed by using the qScript cDNA synthesis kit (Quantabio, Beverly, MA, USA). Real-time PCR was performed using the LightCycler 96 system with LightCycler 480 SYBR Green I Master (Roche Diagnostics, Indianapolis, IN, USA) [17, 82]. The lists of sequences of primers used for quantitative qPCR were provided in Supplementary Table 1. All primers were designed to span an intron to prevent amplification of genomic DNA, and have single-product melting curves, as well as consistent amplification efficiencies between 1.8 and 2.2 [17, 82]. We selected *GAPDH* and *RPS26* as a housekeeping genes, as described previously [16, 82]. The threshold cycle number (Ct) was used to quantify the relative abundance of the gene; arbitrary units were calculated as 2-ΔCt = 2-(Ct target gene - Ct housekeeping gene).

### Statistical analyses - cell culture

Statistical analyses of the in vitro work were performed using GraphPad Prism version 6.01 Software (GraphPad Software, Inc., San Diego, CA). Data are presented as means ± SEM. Cell culture experiments comprised at least four independent repeats; each repeat consisted of cells obtained from different follicles (one follicle/cow), examined in duplicates. Data were analyzed by either Student’s t-test or one-way ANOVA, when indicated. In all analyses, a value of *p* ≤ 0.05 was considered significant.

## Supplementary Information

**Additional file 1 Supplementary Fig. 1.** Principal component analysis (PCA) of P cows and NP cows samples are clustered by gene-expression profile. The analysis was based on the 1000 most variable genes. P; Pregnant cows; NP: Non-pregnant cows.

**Additional file 2 Supplementary Table 1.** List of primers used for qRT-PCR.

## Data Availability

The datasets generated and/or analysed during the current study are available in the GEO repository, [persistent web link to datasets: https://www.ncbi.nlm.nih.gov/geo/query/acc.cgi?acc=GSE163212]. To review GEO accession GSE163212: Go to https://www.ncbi.nlm.nih.gov/geo/query/acc.cgi?acc=GSE163212 Enter token qjurmsqadxcxdcv into the box. The following secure token has been created to allow review of record GSE163212 while it remains in private status.

## Abbreviations

DEGs: Differentially expressed genes
EDN1: Endothelin 1
ECM: Extracellular matrix
GSEA: Gene Set Enrichment Analysis
IPA: Ingenuity Pathway Analysis
PDGF: Platelet Derived Growth Factor
PGF2A: Prostaglandin F2 Alpha
TGFB: Transforming Growth Factor Beta
TNFA: Tumor necrosis factor alpha
