# Supplementary figures and images for: Downregulated luteolytic pathways in the transcriptome of early pregnancy bovine corpus luteum are mimicked by interferon-tau in vitro
Source: BMC Genomics. 2021 Jun 16;22:452. doi: 10.1186/s12864-021-07747-3 (PMC8207607; doi:10.1186/s12864-021-07747-3)

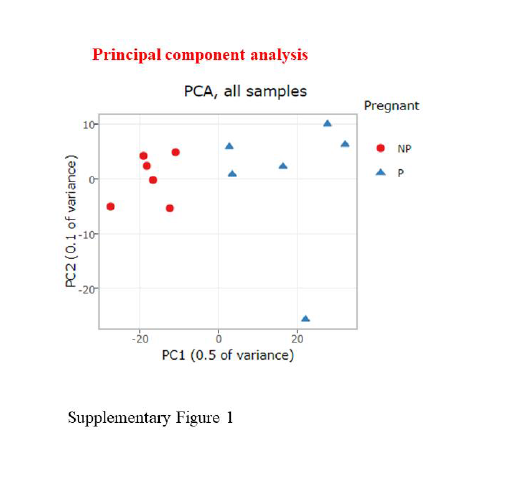

Supplement: Supplementary file 1 — Additional file 1 Supplementary Fig. 1. Principal component analysis (PCA) of P cows and NP cows samples are clustered by gene-expression profile. The analysis was based on the 1000 most variable genes. P; Pregnant cows; NP: Non-pregnant cows. [file 12864_2021_7747_MOESM1_ESM.docx]
