# Supplementary material for: Downregulated luteolytic pathways in the transcriptome of early pregnancy bovine corpus luteum are mimicked by interferon-tau in vitro
Source: BMC Genomics. 2021 Jun 16;22:452. doi: 10.1186/s12864-021-07747-3 (PMC8207607; doi:10.1186/s12864-021-07747-3)
Supplement: Supplementary file 2 — Additional file 2 Supplementary Table 1. List of primers used for qRT-PCR. [file 12864_2021_7747_MOESM2_ESM.docx]

| **Gene Name** | **Primer** | **Sequence (5’-3’)** | **Accession No.** |
| --- | --- | --- | --- |
| *GAPDH* | Forward  Reverse | GTCTTCACTACCATGGAGAAGG  TCATGGATGACCTTGGCCAG | NM_001034034 |
| *GLI1* | Forward  Reverse | CCTTCAAAGCCCAGTACATGC  GTGCGTCTTCAGATTTTCCAG | NM_001099000.1 |
| *GLI2* | Forward  Reverse | TTCACCTCCATCAATGCCACA  TTGACAGTGCTGCTGCTCACGG | NM_001192250.2 |
| *PTCH2* | Forward  Reverse | ACGCCTTCTCCTCTACCAC  CGCAGCATTGTCACACA | XM_005204892.4 |
| *SUFU* | Forward  Reverse | CAGGTTACCGCTATCGTCAAG  CTGTTGTCGCCATAGAGATCG | NM_001098083.2 |
| *TIMP3* | Forward  Reverse | ATCCGAGCCAAGGTGGTAGGG  CCTCAAGCTTAAGGCCACAGA | NM_174473.4 |
| *TGFBR1* | Forward  Reverse | CCAGAGACAGGCCATTTGTATG  GCTGCCAGTTCAACAGGACCAAG | NM_001159566 |
| *TGFBR2* | Forward  Reverse | GCTCCGAAGAATGCCAGAAG  AGTGATGATGACTGCATGG | NM_174621 |
| *ADAM 17* | Forward  Reverse | GCCTTACGTCAACGCCGAAC  GAGGACGGAACCGACGATG | XM_002691486.6 |
| *HPGD* | Forward  Reverse | GAACCTACCTGGGCTTGGATTAC  GCCTGGGCAAATTGCGTTCAGTC | NM_001034419.2 |
| *PTGFR* | Forward  Reverse | TGGGGCACCTTCTTTGATCTGG  AACCTGACAGCCAACCACGTA | NM_181025.3 |
| *THBS2* | Forward  Reverse | GCTTCGTCCGCTTTGACTAC  TAGGTGAGGTCCAGGGTGT | NM_176872 |
| *MMP9* | Forward  Reverse | GAGAGGGTCGCAATGATG  CTGGCACGGAGGTGTGATCTA | NM_174744 |
| *BMP4* | Forward  Reverse | ACTGGGATCAGGGCTTTCATC  CTCACATCAAAAGTTTCCCACCG | NM_001045877.1 |
| *PTGS2* | Forward  Reverse | CAGCGGTGCAGCAAATCCTTG  CTGTGTTGGGAGTGGGTTTCA | NM_174445 |
| *RPS26* | Forward  Reverse | CCAACTGTGCCCGATGTG  TTCCCGAGAGCGATTCCTGA | NM_001015561 |

**Supplementary Table 1. List of primers used for qRT-PCR.**
